# Comparative study of the effect of rice husk-based powders used as physical conditioners on sludge dewatering

**DOI:** 10.1038/s41598-020-74178-7

**Published:** 2020-10-14

**Authors:** Maoqing Wang, Yan Wu, Binrong Yang, Peiyao Deng, Yinhai Zhong, Chuan Fu, Zenghui Lu, Panyue Zhang, Jueqiao Wang, Yuyang Qu

**Affiliations:** 1 Key Laboratory of Water Environment Evolution and Pollution Control in Three Gorges Reservoir, Chongqing Three Gorges University, 404100, Wan Zhou, Chongqing, People’s Republic of China; 2 College of Environmental Science and Engineering, Beijing Forestry University, Beijing, 100083 People’s Republic of China; 3 Jiangsu Tian Hong Environmental Engineering Co., Ltd, Yangzhou, 225000 People’s Republic of China; 4 Chongqing Wanzhou Ecological Environmental Monitoring Station, 404100, Wan Zhou, People’s Republic of China

**Keywords:** Sustainability, Pollution remediation, Environmental biotechnology

## Abstract

The effects of rice husk flour (RHF), rice husk biochar (RHB), and rice husk-sludge cake biochar (RH-SCB, expresses sludge cake biochar deriving from a sludge that has been previously conditioned with rice husk) used as physical conditioners on sludge dewaterability were compared. The effects of characteristics of physical conditioners on sludge compressibility and zeta potential were analyzed. The optimal rice husk-based powder was RH-SCB, which presented the highest net sludge solid yield (Y_N_, expresses the dry mass flow by filtration) at 20.39 kg/(m^2^ h) for 70% dry sludge (DS). Characterization analysis indicates that the hardness and surface Fe content of powders which could influence the compressibility coefficient of sludge cake and sludge zeta potential were the major factors influencing sludge dewaterability. The comparison of feasibility and economic analysis showed that adding RH-SCB improves the quality of the sludge filtrate and reduces the pollution potential of conditioned sludge (the ratio of secondary and primary (RSP) of Cu, Zn, Cd reduces from 43.05, 144.00, 7.25 to 7.89, 14.63, 4.27, respectively), and the costs of using RH-SCB were the lowest (at 88.4% lower than that of the raw sludge). Therefore, it is feasible to use RH-SCB to improve sludge dewaterability.

## Introduction

Most municipal wastewater treatment plants (WWTPs) use activated sludge to treat wastewater^1^. Due to the increasing amount of wastewater and current efficiency of wastewater treatments, the amount of sewage sludge (moisture content higher than 90%) produced worldwide has also increased. This sludge must be treated and disposed appropriately because it can contain several pollutants that were removed from the wastewater^2,3^. In this context, it is still a challenge to efficiently treat domestic and industrial wastewaters.

Sludge normally contains a significant proportion of water^1^, which hinders the use of disposal methods such as composting and incineration. Thus, sludge conditioning and dewatering are essential for its treatment^4,5^. Currently, a common technique for dewatering of sewage sludge is mechanical dewatering. However, this method can be costly due to the low filtration rate of sludge and the use of high mechanical pressure^6^. Therefore, sludge dewatering must be effectively improved. Chemical flocculants such as cationic polyacrylamide (CPAM) and ferric chloride (FeCl_3_) are commonly used in WWTPs to separate water from sludge and improve the efficiency of sludge dewatering by charge neutralization and adsorption bridge^7,8^. However, the final steps of sludge dewatering are difficult to perform due to the formation of a compact sludge cake^9,10^.

To solve this problem, physical conditioners are used in sludge dewatering as they can improve the structure permeability of sludge cakes^11^. The use of waste biomass-based materials has been investigated for this purpose^12^. The waste biomass-based materials include raw waste biomass-based materials flour (such as rice husk flour, bamboo flour^13^, and wood sawdust^14^, etc.), biomass-based biochar (such as rice husk biochar^15^), and sludge cake biochar conditioned by biomass-based materials (the sludge cake biochar derived from pyrolysis of dewatered sludge which was conditioned with different biomass-based materials such as rice husk, bamboo, and wood, etc.). All of these materials lead to low compressibility of the sludge cake during dewatering under high pressure. A permeable skeleton structure is formed in the sludge cakes, thus increasing the removal of water and, consequently, the sludge dewaterability. However, it is not clear what kind of waste biomass-based material is better for sludge dewatering, raw flour, biochar, or sludge cake biochar conditioned by biomass-based materials. It was reported that the dewaterability of sludge conditioned with sawdust and cationic polyacrylamide (CPAM) was superior to the dewaterability of sludge conditioned with CPAM alone^14^. And Wang et al. also found that the SRF and CST of sludge conditioned with bamboo flour and rice husk flour was lower than those of sludge conditioned without these skeleton builders^13^. The above studies showed that biomass-based materials could further increase sludge dewaterability. But few studies have compared the effects of different waste biomass-based materials on sludge dewaterability. Moreover, the feasibility of adding different waste biomass-based materials including the effects of different waste biomass-based materials on the pollution potential of heavy metals in the sludge, and pH and SCOD of sludge filtrate which can significantly influence sludge disposal, and the disposal costs have not been compared.

In our previous experiment, rice husk-based powders, such as rice husk flour, rice husk biochar, and sludge cake biochar conditioned by rice husk flour, are used as physical conditioners to improve sludge dewaterability^6,16^. In this study, the effects of different rice husk-based physical conditioners on sludge dewaterability were compared. Characteristics such as microstructure, surface element content, component analysis, surface zeta potential, and specific surface area of rice husk-based powders were analyzed to clarify the mechanisms of improving dewaterability. The transference and translation of heavy metals and their pollution levels in the sludge, in addition to the pH and SCOD of sludge filtrate and the disposal costs, were compared. The results provide a theoretical basis for choosing proper physical conditioners.

## Materials and method

### Materials

#### Physical conditioners

Following our preliminary study^17^, rice husk flour (RHF, 109–150 μm) was produced from rice husk upon grounding and sieved by 100-mesh and 140-mesh screen. Rice husk biochar (RHB, 80–250 μm) was prepared at 500 °C for 2 h in an electric tube furnace (SK2-2-13, China) under nitrogen protection, sieved by 60-mesh and 180-mesh screen. After the sludge conditioned with FeCl_3_ (115.07 g/kg DS) and rice husk flour (0.7 kg/kg DS), the conditioned sludge was dried and then put into an electric tube furnace under the protection of nitrogen prepared at 400 °C for 2 h. Finally, the sludge cake biochar deriving from a sludge that has been previously conditioned with rice husk (0.7 kg/kg DS) and FeCl_3_ (115.07 g/kg DS) (Rice husk-sludge cake biochar, RH-SCB, 80–250 μm) was obtained. For that, a FeCl_3_ solution (5 g/L), which is commonly used as an inorganic conditioner, was used.

#### Raw sewage sludge

For the experiments, the sample of raw sewage sludge from a local municipal WWTP of Chongqing, China, was used. Before the treatment, the samples were stored in sealed plastic buckets at 4 °C in a laboratory refrigerator to reduce the activity of microorganisms^18^. Before each experiment, the sludge was heated for 30 min at 20 °C in a thermostatic water bath to ensure compliance with the actual sludge treatment in the sewage treatment plant^16^. The main characteristics of raw sewage sludge are shown in Table 1. The Y_N_ used to evaluate the sludge filterability in this work, represents the mass of sludge solids filtered per unit area and unit time, which can be calculated according to Ning et al.^19^.

**Table 1:** Main characteristics of raw sewage sludge.

Moisture content (%)	Dry sludge (DS) (g/L)	SRF (m/kg)	Y_N_ (kg/(m^2^ h))
99.0–99.2	8.38–8.46	3.66 × 10^13^–4.03 × 10^13^	0.90–0.95

### Sludge conditioning and dewatering

Rice husk-based powders and FeCl_3_ (115.07 g/kg DS) were added in sequence into the sewage sludge. After a mixing apparatus (JJ-6, China) was used to stir the mixture, 100 ml of the sample was poured into a Buchner funnel (8 cm), and then filtered at a filter pressure of 0.03 MPa. The net sludge solids yield (Y_N_) was used as the main evaluating index of sludge dewaterability. Specific resistance to filtration (SRF) and moisture content of filtered sludge cake were used as secondary indexes. Additionally, the compressibility coefficient of sludge cakes conditioned with different rice husk-based powders was measured. The final value for each experiment was the average result of two to three repeated tests.

### Transference and translation of heavy metals and their concentration in the sludge

In our previous studies, the main heavy metals observed in sludge were Cu, Zn, and Cd. Therefore, their contents in the raw and conditioned sludge filtrate were measured. Moreover, the content and five fractions (namely exchangeable, carbonate-bound, Fe–Mn oxide-bound, organic matter-bound, and residual) of heavy metals in the raw and conditioned sludge cake were tested to analyze the transference and translation of heavy metals. The content of heavy metals was measured by an inductively coupled plasma optical emission spectrometer (ICP-OES) (Optima 7000, USA), and the five fractions of heavy metals were measured by a sequential extraction procedure according to Fu Chuan^20^. The pollution level of heavy metals in sludge cakes was reflected with the ratio of secondary and primary phases (RSP) calculated by Li^21^. The calculation formula of RSP is shown in Text S1.

### Characteristics of physical conditioners

The surface zeta potential of the physical conditioners was tested by using a Zetasizer Nano analyzer (ZEN3600, England). An environmental scanning electron microscope (ESEM; Quanta 200, America) was used to test the microstructures of the rice husk-based powders. Specific surface areas and component analysis were investigated by a specific surface area analyzer (ChemiSorb 2720, America) and X-ray diffractometer (XRD) (D5000, Germany), respectively. Surface element contents were analyzed by energy-dispersive spectrometry (EDS) (EDAX genesis XM-2, America).

### Analytical methods

Sludge SRF was measured by the Buchner funnel method^22^, moisture content of sludge cake was tested by a gravimetric method, and Y_N_ was calculated by SRF, according to Ning et al.^19^. These methods were combined to evaluate the sludge filterability and dewaterability^23^. The compressibility coefficient of sludge cakes conditioned with different rice husk-based powders was calculated by sludge SRF under different filtration pressures^24^. The pH of the sludge filtrates was tested by an acidimeter (PHS-3C, China). The filtrate SCOD was measured according to the potassium dichromate method after filtering through a 0.45 μm membrane^25^.

## Results and discussion

### Effects of different rice husk-based powders on sludge dewaterability

**Figure 1:**
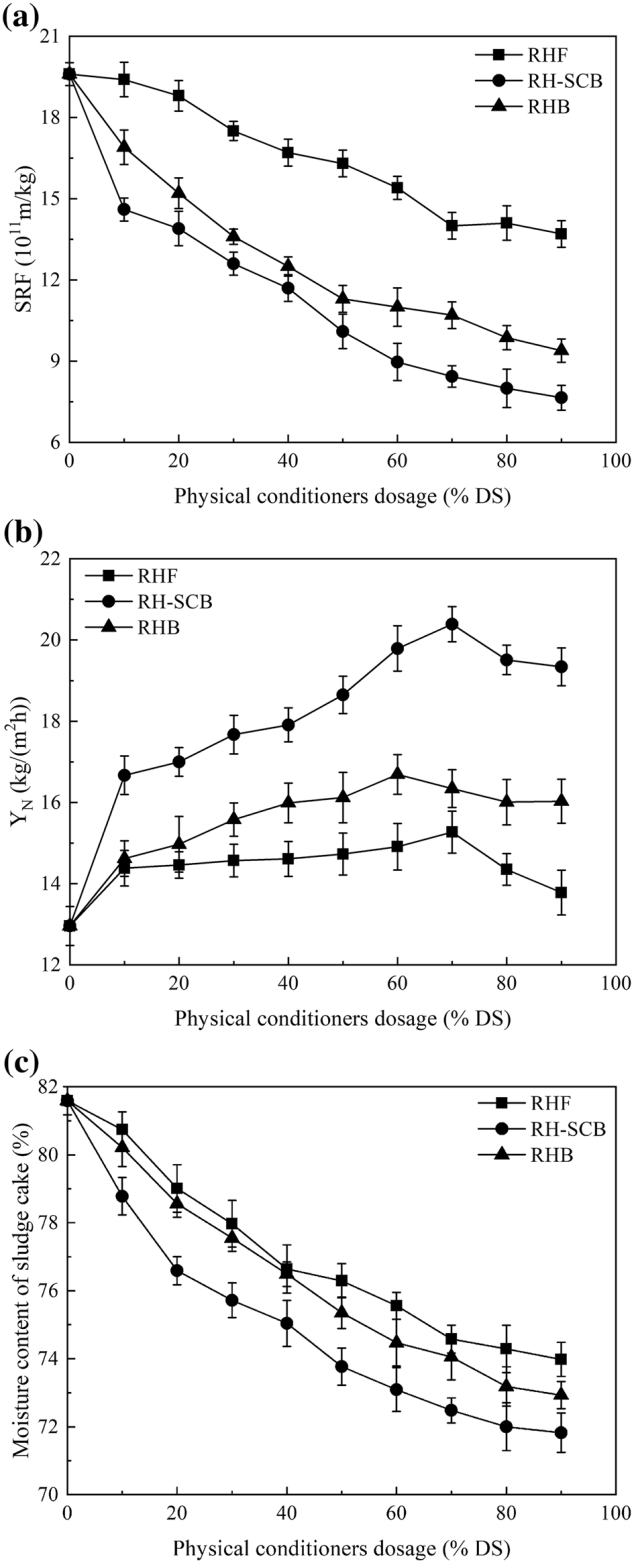
Effect of different rice husk-based powders as physical conditioners on sludge dewaterability (**a**) SRF, (**b**) Y_N_ and (**c**) moisture content of sludge cake (FeCl_3_ dosage of 115.07 g/kg DS).

Figure 1 shows the effects of different rice husk-based powders used as physical conditioners on sludge dewaterability. The sludge Y_N_ increased with increasing powder dosage, while the sludge SRF and moisture content of sludge cakes decreased. The Y_N_ upon the use of rice husk-sludge cake biochar (RH-SCB) was higher than those upon the use of rice husk flour or biochar. Moreover, the SRF and moisture content were the lowest for the sludge cake conditioned with RH-SCB. The highest Y_N_ of 20.39 kg/(m^2^ h) was achieved when the dosage of RH-SCB was 70% dry sludge (DS) (0.7 kg RH-SCB/kg DS). These results indicate that, among the studied materials, RH-SCB is the optimal one for use as a physical conditioner to enhance sludge dewaterability. The reason may be that the surface iron content of RH-SCB is high, and the surface charge of the RH-SCB is positive, causing the RH-SCB to embed in flocs by charge neutralization with sludge particles before adding FeCl_3_ and work better as a skeleton builder during sludge dewatering^17^.

### Effects of different rice husk-based powders on sludge cake compressibility

**Figure 2:**
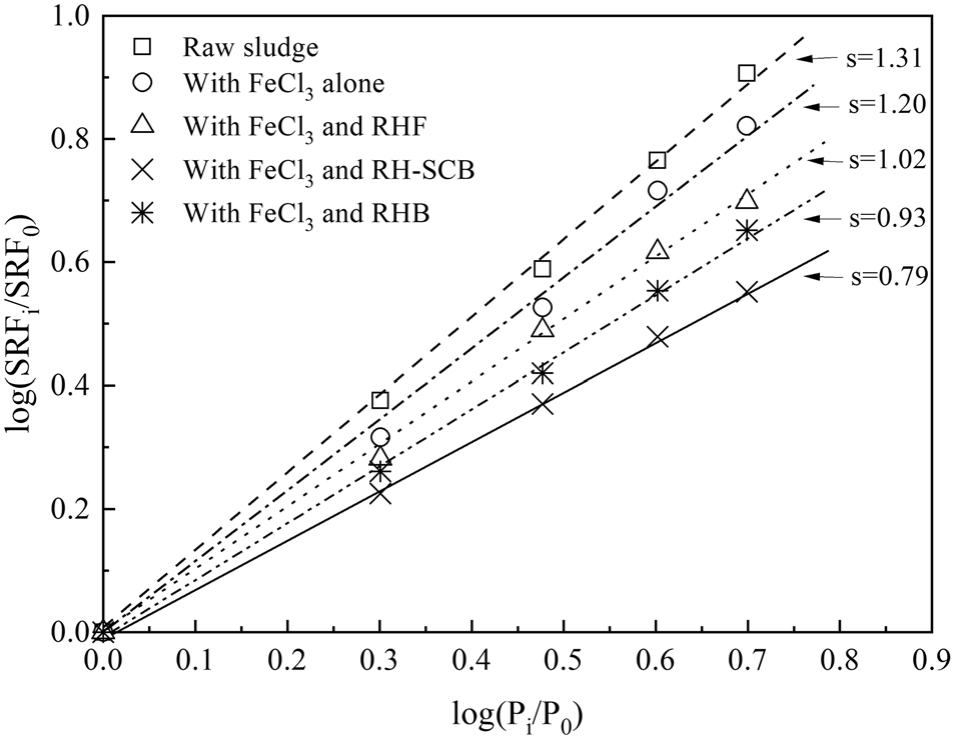
Coefficient of compressibility of sludge cakes (FeCl_3_ dosage of 115.07 g/kg DS, RHF dosage of 70% DS, RH-SCB dosage of 70% DS, and RHB dosage of 60% DS).

Reducing sludge cake compressibility is the main function of physical conditioners in sludge conditioning and dewatering^4^. Figure 2 shows the compressibility of sludge cake conditioned with different rice husk-based powders. All the compressibility coefficients were lower than those of raw sludge cake and sludge cake with FeCl_3_ alone. When the RH-SCB was used, the sludge cake compressibility (s = 0.79) was the lowest, which means that the obtained sludge cake was the most permeable and its sludge dewaterability was the best out of the different options tested^17^. These results indicated that RH-SCB plays a better support role during sludge dewatering which might be caused by the high content of surface iron of RH-SCB. Therefore, the RH-SCB, deriving from a sludge that has been previously conditioned with FeCl_3_ (115.07 g/kg DS) and rice husk flour (0.7 kg/kg DS), was the best option for use as a sludge physical conditioner in this study. The obtained results are consistent with those shown in Fig. 1.

### Effects of different rice husk-based powders on sludge zeta potential

The zeta potential of the sludge with different rice husk-based powders are shown in Table S1. Only the surface charge of the RH-SCB was positive among all three rice husk-based powders. When only the RH-SCB was used, the zeta potential of the sludge was closer to 0 mV, which implies that the sludge colloids attracted each other and formed an unstable system that caused good settling and dewatering performance^4^. Both surface charge of RHF and RHB are negative, and they could not flocculate with sludge particles before adding FeCl_3_ and might be on the outside of sludge flocs with adding FeCl_3_. The flocs with adding RHF or RHB were still compact during sludge dewatering. But RH-SCB could flocculate with negatively-charged sludge particles and embedded in flocs before adding FeCl_3_. The flocs with adding RH-SCB were permeable and more moisture of sludge could be removed. These results prove the inference in Fig. 1, and they indicate that the surface zeta potential significantly impacts sludge conditioning and dewatering.

### Characterization analysis

Characteristics such as microstructures, specific surface areas, surface Fe content, and component analysis of the different rice husk-based powders were tested to analyze the mechanism of improving dewaterability. The microstructures (Fig. S1) indicate that the RH-SCB contained rice husk-based biochar and sludge-based biochar^17^. The surface of the RHB was more porous and wrinkled than those of RHF and sludge-based biochar. The specific surface area (Table S2) was higher for RH-SCB (25.012 m^2^/g) compared to RHF (2.473 m^2^/g), but lower compared to RHB (56.032 m^2^/g). Therefore, the specific surface area of rice husk-based powders was not the major influencing factor for sludge conditioning and dewatering.

The component analysis (Fig. S2) shows that RH-SCB contains large amounts of silica, which may have caused the strongest hardness and greatest support of RH-SCB (RH-SCB could not be crushed under high pressure during sludge dewatering), consequently leading to the lowest compressibility coefficient of sludge cake (consistent with the results of Fig. 2). In addition, according to the Fe contents (Table S3), the highest content of iron species was observed on the surface of RH-SCB. Fig. S2 also indicates that the RH-SCB contains iron compounds such as NaFeS_2_•2H_2_O, KFe_2_P_2_, and Fe_3_(PO_4_)_2_·8H_2_O, which are not present in rice husk flour and biochar. These iron species derive from the existent FeCl_3_ and are formed in the preparation of the RH-SCB. These iron species on the surface of the RH-SCB lead to a positive charge, thereby resulting in optimal surface zeta potential and dewaterability of the sludge that was conditioned with FeCl_3_ (115.07 g/kg) and RH-SCB (70% DS)^26^. To sum up, the RH-SCB was hard, so they could not be crushed under high pressure during sludge dewatering and play a better support role in sludge cake; the surface charge of the RH-SCB was positive, so they could embed in flocs before adding FeCl_3_ and improve the flocs permeability, resulting in more moisture of sludge being removed. These results indicate that the hardness and content of the surface Fe element of rice husk-based powders can influence the sludge cake compressibility and sludge zeta potential. Therefore, they are the major influencing factors for sludge conditioning and dewatering.

### Comparison of feasibility and economic analysis

#### Transference and translation of heavy metals in sludge

**Figure 3:**
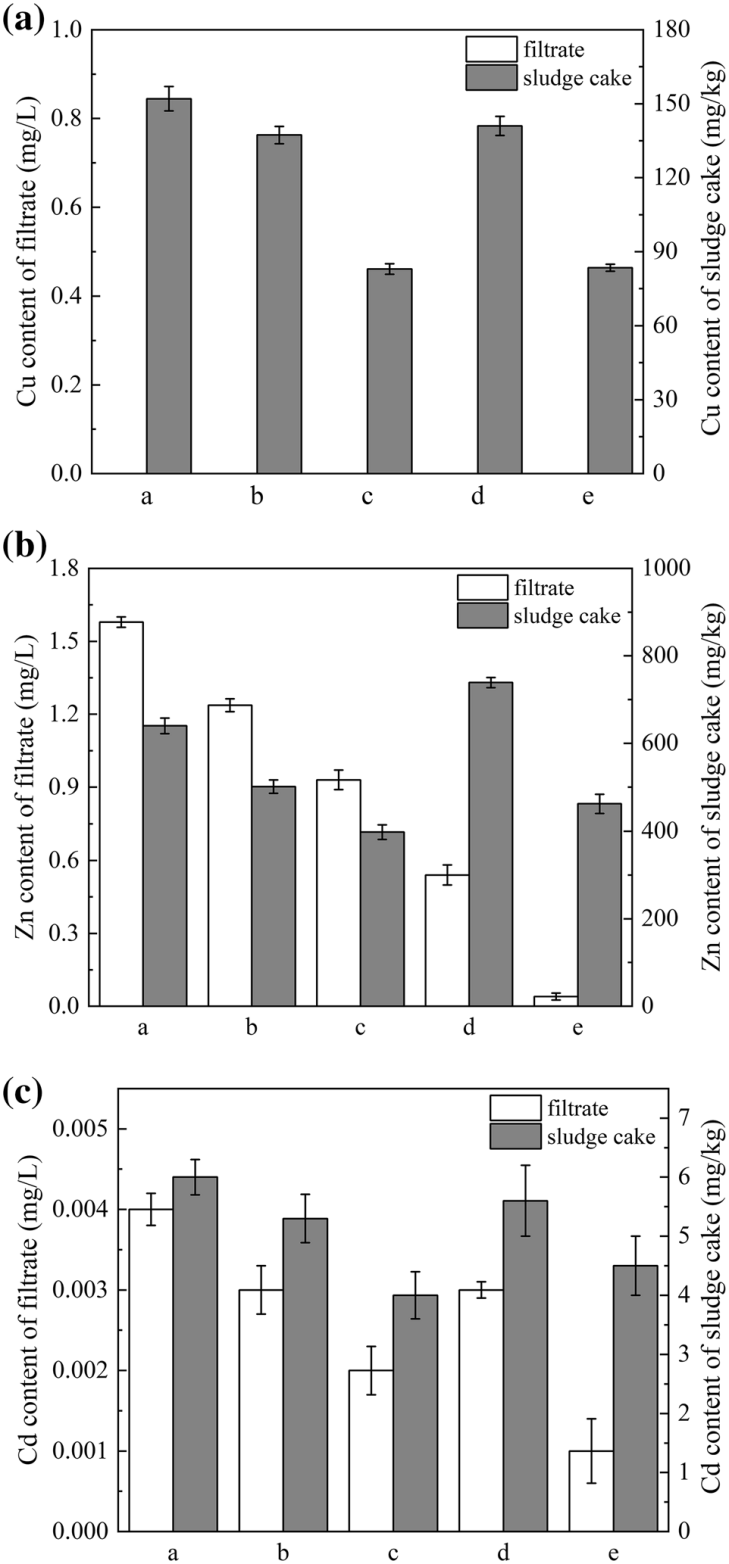
Heavy metals contents of sludge filtrate and sludge cakes (FeCl_3_ dosage of 115.07 g/kg DS, RHF dosage of 70% DS, RH-SCB dosage of 70% DS, and RHB dosage of 60% DS). a-raw sludge, b-sludge conditioned by FeCl_3_ alone, c-sludge conditioned by RHF, d-sludge conditioned by RH-SCB, e-sludge conditioned by RHB.

The heavy metal content in the raw and conditioned sludge filtrates and the content of five fractions of heavy metals in the raw and conditioned sludge cake were tested to analyze the transference and translation of heavy metals in the sludge. Figure 3 shows the heavy metals contents of sludge filtrates and cakes. As shown in Table S4, the heavy metal content of all sludge filtrates met the Chinese discharge standards, except for Zn in the raw sludge filtrate. All sludge cakes met the Chinese standards for grade B sludge agricultural products. The content of heavy metals in the sludge filtrate and cakes conditioned by RHF, RH-SCB, and RHB were lower than those in raw sludge filtrate, except for Zn after conditioning by RH-SCB. The heavy metals contents of the sludge filtrate and cake conditioned by RHB were the lowest, likely because the RHB has a strong adsorption capacity at the maximum specific surface area (Table S2). The heavy metals contents of sludge cake conditioned by RH-SCB were the highest, likely because the RH-SCB was prepared from a part of sludge, which contains a high content of heavy metals (Table S5). Also, the dry sludge cake of unit mass of the sludge conditioned by rice husk flour and rice husk biochar contained a certain amount of dry sludge and skeleton particles. It can be seen from Table S5 that rice husk flour and rice husk biochar contain relatively low heavy metal content. But in RH-SCB, the content of the three heavy metals is much higher than RHF and RHB (according to Table S5). So the content of three heavy metals in the sludge cake after the combined conditioning of RH-SCB and FeCl_3_ is higher. Table S6 shows the total mass of heavy metals of sludge filtrates and cakes. And the results in Table S6 indicate that the total mass of heavy metals in sludge has not changed.

**Figure 4:**
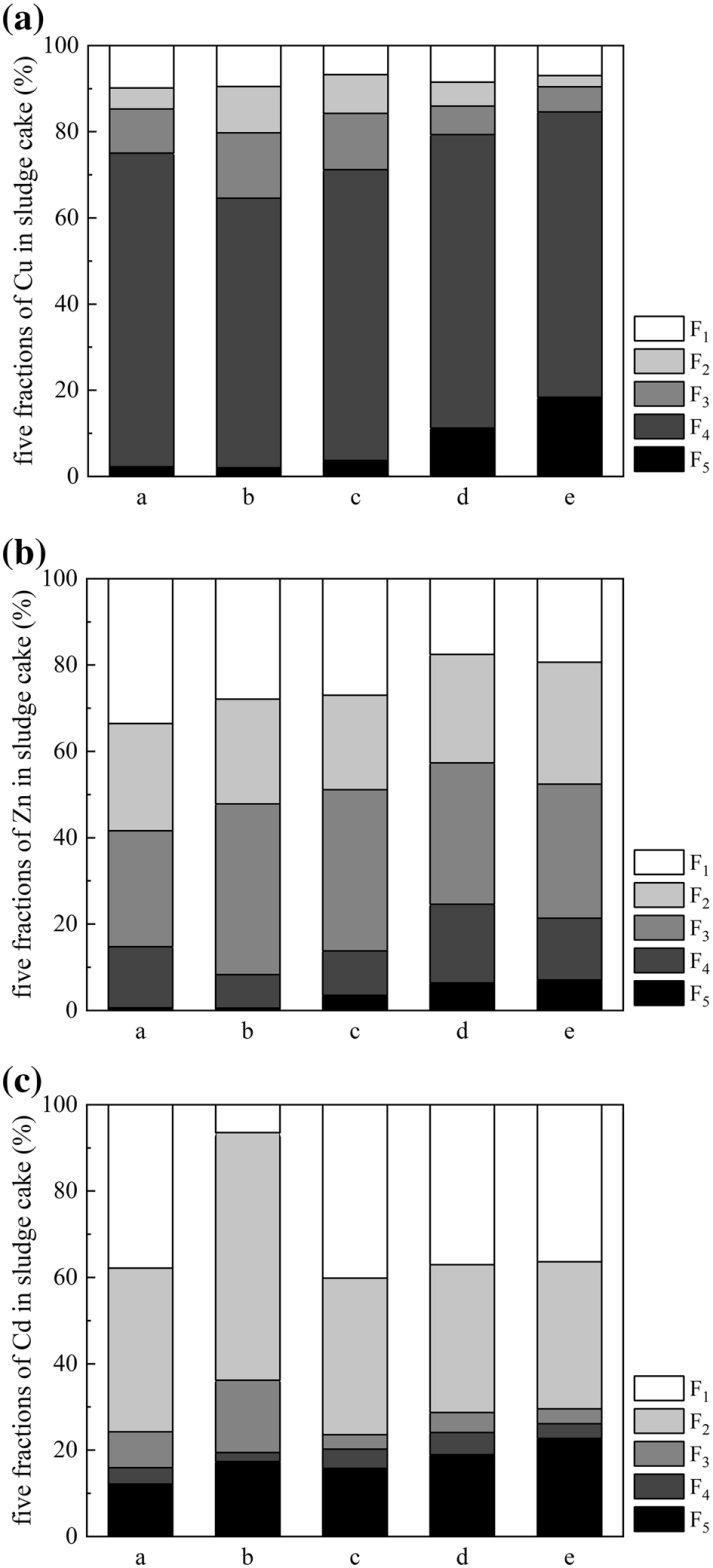
Five fractions of heavy metals in sludge cakes (FeCl_3_ dosage of 115.07 g/kg DS, RHF dosage of 70% DS, RH-SCB dosage of 70% DS, and RHB dosage of 60% DS). a-raw sludge, b-sludge conditioned by FeCl_3_ alone, c-sludge conditioned by RHF, d-sludge conditioned by RH-SCB, e-sludge conditioned by RHB, F_1_—exchangeable, F_2_—carbonate-bound, F_3_—Fe–Mn oxide-bound, F_4_—organic matter-bound, F_5_—residual.

Figure 4 shows the five fractions of heavy metals in the sludge cakes. Table S7 shows the respective RSP values, which indicate the risks posed by heavy metals in different sludge cakes. All RSP values of sludge cakes conditioned by rice husk-based powders were lower than that of raw sludge cake (Cu (43.05), Zn (144.00), Cd (7.25)) and sludge cake conditioned by FeCl_3_ alone (Cu (48.07), Zn (173.88), Cd (8.01)), and the RSP of sludge cakes conditioned by RHB were the lowest (Cu (4.45), Zn (13.18), Cd (3.39)). Although the heavy metal contents of sludge cakes conditioned by RH-SCB were high, the RSP values (Cu (7.89), Zn (14.63), Cd (4.27)) were still lower, which indicates that the heavy metals in the sludge cake conditioned by RH-SCB presented a lower pollution potential than those in the raw sludge cake and sludge cake conditioned by FeCl_3_ alone.

#### SCOD and pH of sludge filtrate

As shown in Fig. S3, the addition of RHF, RHB, and RH-SCB elevated the pH of the filtrate compared with FeCl_3_ conditioning, thereby reducing the corrosion of the filtrate on the elevated dewatering equipment. The SCOD of the sludge filtrate conditioned by RH-SCB was significantly lower than that of the other samples, thereby reducing the cost of filtrate reprocessing. This result indicates that sludge dewatering conditioned by RH-SCB does not deteriorate the water quality of the filtrate and is feasible from an economic perspective.

#### Economic analysis

An economic analysis of the raw sludge dewatering and sludge conditioning by FeCl_3_ alone, RHF (70% DS), RHB (60% DS), and RH-SCB (70% DS) was performed, and it considered materials, reagents, and final disposal costs. The energy requirement for the pyrolysis of rice husk biomass was determined as 0.3 MJ/kg dry rice husk^27^, dried sludge 0.15 MJ/kg DS^28^, and the energy consumed for the preparation of RHB and dried sludge cake was calculated following Gil-Lalaguna^28^. The selling price of RH-SCB is assumed to be 0.04 USD/kg^29^. The CaO is used to stabilize the dewatered sludge, except for the dewatered sludge conditioned by RH-SCB, and the dosage is assumed to be 25% of the wet sludge^30^. The prices and other economic parameters are shown in Table S8. According to the calculations, the disposal costs of raw sludge and sludge conditioned by FeCl_3_ alone, RHF, RHB, and RH-SCB were 7.07, 0.94, 1.29, 1.59, and 0.82 USD/kg DS, respectively. Therefore, RH-SCB conditioning was the most economical disposal method, as it represented an 88.4% cost reduction compared with the disposal of raw sludge.

## Conclusions

From all analyzed options, the RH-SCB (rice husk-sludge cake biochar), deriving from a sludge that has been previously conditioned with FeCl_3_ (115.07 g/kg DS) and rice husk flour (0.7 kg/kg DS), was the best option for use as a physical conditioner to condition and dewater sludge in this work. The Y_N_ (20.39 kg/(m^2^ h)) was the highest for 115.07 g/kg of FeCl_3_ and 70% DS RH-SCB. The sludge cake compressibility (s = 0.79) was the lowest and the zeta potential of the sludge was the closest to 0 mV when RH-SCB was used. The characterization analysis indicated that the surface zeta potential, hardness, and surface Fe content of rice husk-based powders were the major factors influencing sludge conditioning and dewatering. In addition, sludge conditioning with RH-SCB improved the water quality of the sludge filtrate and reduced the risk of heavy metals in the sludge cake and the costs of treatment and disposal. However, this economic analysis was based on the expansion of experimental data, and it does not represent the result of an actual application. As a whole, the RH-SCB used as a latent physical conditioner to improving dewaterability is economical and feasible.

## Supplementary information

Supplementary Information.
