# Supplementary material for: Comparative study of the effect of rice husk-based powders used as physical conditioners on sludge dewatering
Source: Sci Rep. 2020 Oct 14;10:17230. doi: 10.1038/s41598-020-74178-7 (PMC7560731; doi:10.1038/s41598-020-74178-7)
Supplement: Supplementary file 1 — Supplementary Information. [file 41598_2020_74178_MOESM1_ESM.pdf]

Supplementary Materials for

Comparative study of the effect of rice husk-based powders used as physical conditioners on sludge dewatering

Maoqing Wang[a], Yan Wu[a, b, c*], Binrong Yang[a], Peiyao Deng[a], Yinhai Zhong[a], Chuan Fu[a**], Zenghui Lu[d], Panyue Zhang[a, b**], Jueqiao Wang[a], Yuyang Qu[a]

[a] Key Laboratory of Water Environment Evolution and Pollution Control in Three Gorges Reservoir (Chongqing Three Gorges University), Wan Zhou 404100, P. R. China

[b] College of Environmental Science and Engineering, Beijing Forestry University, Beijing 100083, P. R. China

[c] Jiangsu Tian Hong Environmental Engineering Co., Ltd. Yangzhou 225000, P. R. China

[d] Chongqing Wanzhou Ecological Environmental Monitoring Station, Wan Zhou 404100, P. R. China

**Corresponding author:* Yan Wu, Chuan Fu, Panyue Zhang

Tel.: +86 18905591879; Fax: +86 23 58102063.

E-mail address: wuyan19850827@hotmail.com (Yan Wu)

(Total 2 texts, 3 figures, and 8 tables)

Text S1 ratio of secondary phase and primary phase (RSP) could be calculated as follows:

$$RSP=M_{sec}/M_{prim}=(F_1+F_2+F_3+F_4)/F_5$$

where:

M_{sec} is the secondary phase and M_{prim} is the primary phase (Li et al., 2019). F_1, F_2, F_3, and F_4 are the concentrations of exchangeable, carbonate-bound, Fe-Mn oxide-bound, organic matter-bound, respectively, and F_5 is the concentration of the residual.

Text S2 The energy needed for drying rice husk and sludge could be calculated as follows:

$$Q_{drying}=\left(m_{dried}Cp_{ss}+m_{H_2O}Cp_{H_2O}\right)\Delta T+m_{H_2O,evap}\Delta H_{vap,H_2O}$$

where:

- Q_{dring} is the heat needed for drying rice husk or sludge cake step (MJ);
- m_{dried} is the dried mass of the dewatered rice husk or the sludge cake (kg);
- m_{H_2O} is the mass of water in the dewatered rice husk or sludge cake (kg);
- ΔT is the difference temperature between the beginning and the end stage of the drying rice husk or sludge process (from 25 to 105 ℃);
- Cp_{ss} is the heat capacity of the biomass or the dried sludge cake. The heat capacity of the biomass assumed to be 1.0×10^{-3} MJ $kg^{-1}K^{-1}$ (Manganaro et al., 2011), and the heat capacity of dried sludge cake was experimentally obtained at 25 °C (1.15×10^{-3} MJ $kg^{-1}K^{-1}$)(Gil-Lalaguna et al., 2014). The value was considered constant during pyrolysis step;
- Cp_{H_2O} is the heat capacity (Gil-Lalaguna et al., 2014) for liquid water (4.18×10^{-3} MJ $kg^{-1}K^{-1}$);
- $m_{H_2O,evap}$ is the mass of water evaporated from the rise husk or the dewatered sludge cake (kg);
- $\Delta H_{vap,H_2O}$ is the enthalpy of vaporization of water (Gil-Lalaguna et al., 2014) at the exit temperature (2.26 MJ kg^{-1} at 100 ℃).

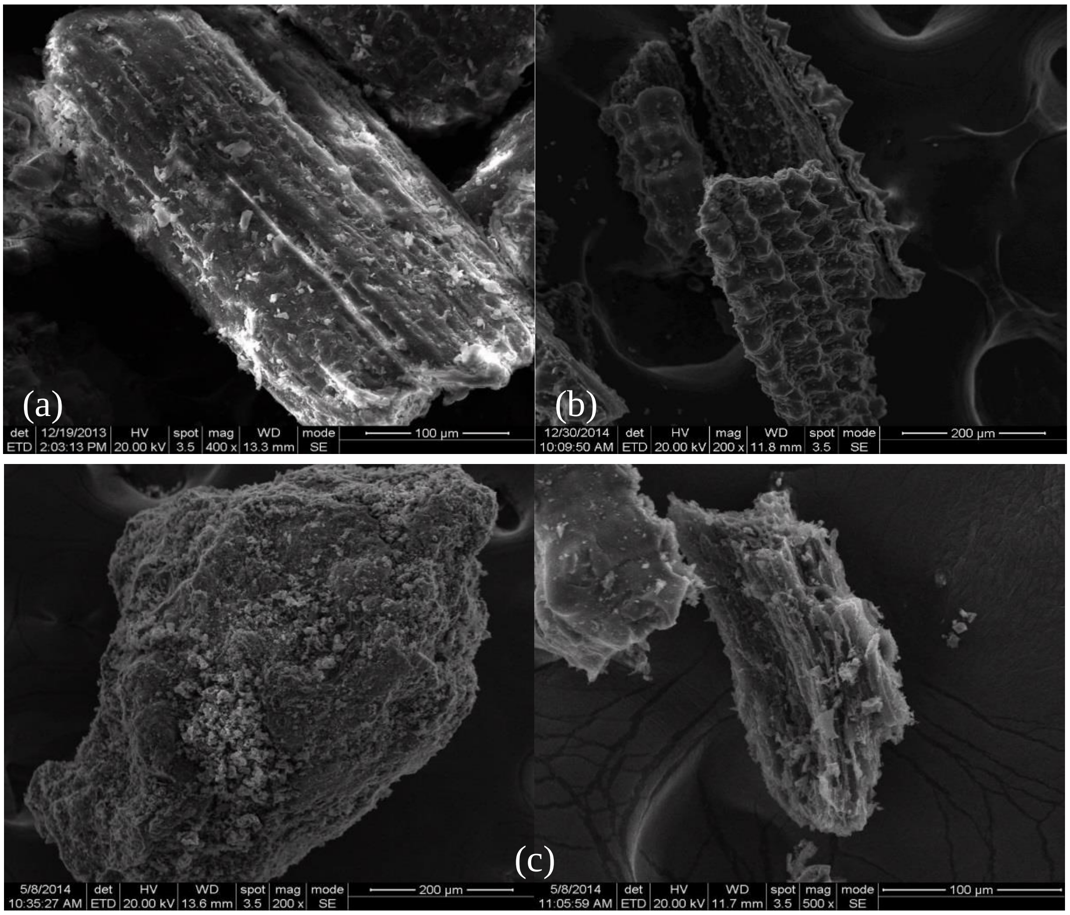

Fig. S1 Microstructures (a) RHF, (b) RHB and (c) RH-SCB.

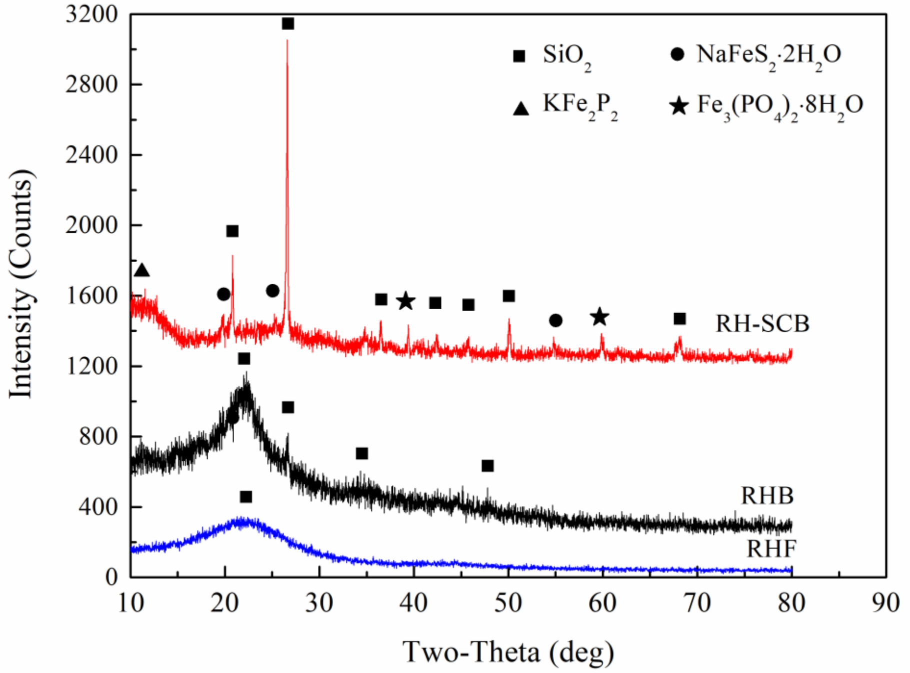

Fig. S2 Component analysis of different Rice husk-based powders as physical conditioners tested by XRD.

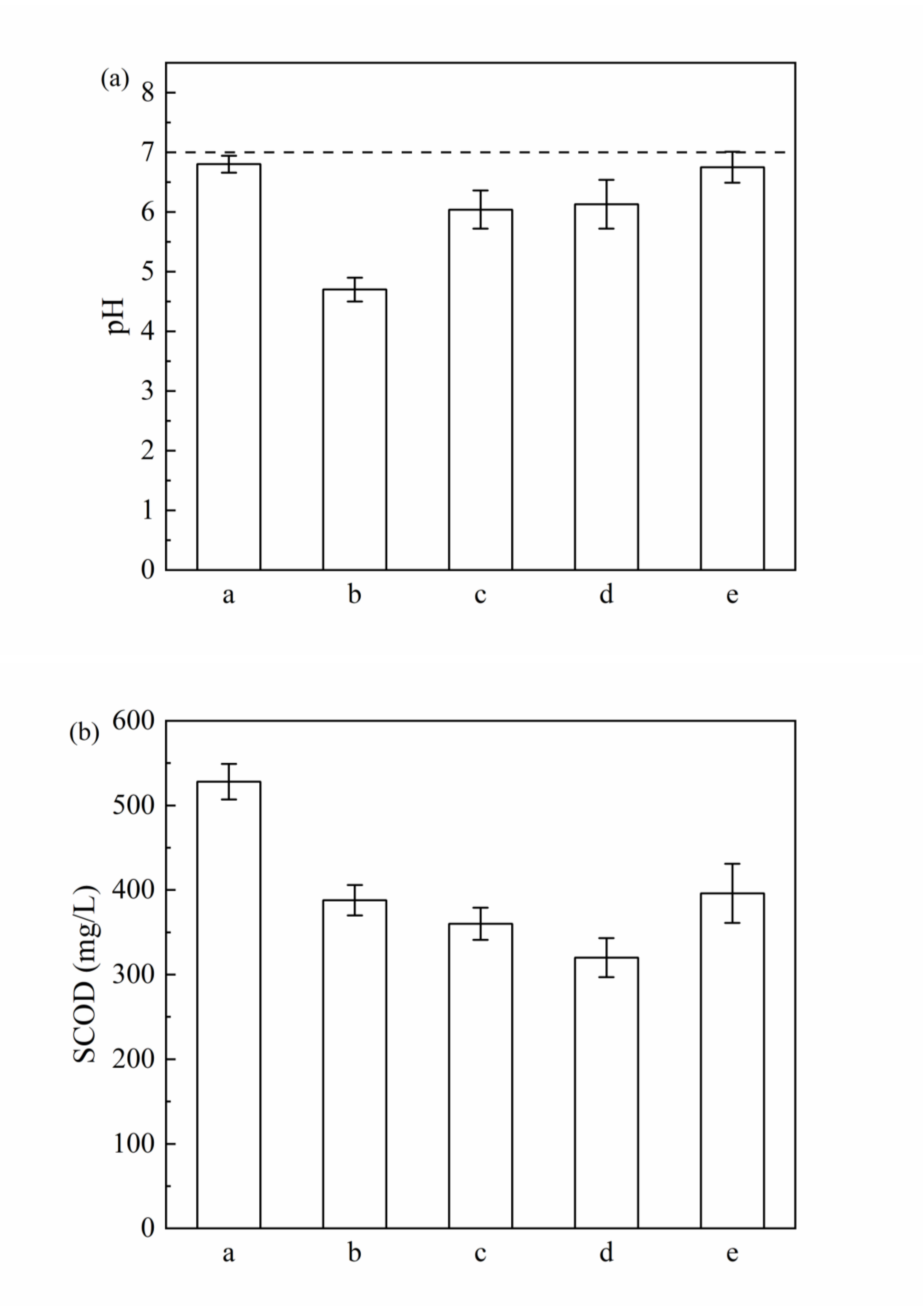

Fig. S3 The pH and SCOD of filtrate (a-raw sludge, b-sludge conditioned by $FeCl_3$ alone, c-sludge conditioned by RHF, d-sludge conditioned by RH-SCB, e-sludge conditioned by RHB)

Table S1 Zeta potential.

	Zeta potential (mV)
RHF (70% DS)	-25
RHB (60% DS)	-28.60
RH-SCB (70% DS)	2.91
Raw sludge	-14.1
Sludge conditioned with $FeCl_3$ (115.07 g/kg) alone	-2.28
Sludge conditioned with $FeCl_3$ (115.07 g/kg) & RHF (70% DS)	-7.17
Sludge conditioned with $FeCl_3$ (115.07 g/kg) & RHB (60% DS)	-9.22
Sludge conditioned with $FeCl_3$ (115.07 g/kg) & RH-SCB (70% DS)	1.18

Table S2 Specific surface area of different Rice husk-based powders as physical conditioners.

	Specific surface area (m^2/g)
RHF	2.473
RH-SCB	25.012
RHB	56.032

Table S3 Surface Fe element content of different Rice husk-based powders as physical conditioners tested by EDS (Wt %).

	Surface Fe element content EDS (Wt. %)
RHF	0.07
RH-SCB	6.25
RHB	0.36

Table S4 The discharge Chinese standards of heavy metals contents of all sludge filtrate and sludge cakes

Chinese standard		Unit	Cu	Zn	Cd
Discharge Standard Of Pollutants For Municipal Wastewater Treatment Plant (GB 18918-2002)		mg/L	0.5	1.0	0.01
Control Standards For Pollutants In Sludges From Agriculture Use (GB 4284-2018)	Grade B sludge products	mg/kg	<1500	<3000	<15

Table S5 Heavy metals contents of Rice husk-based powders (mg/kg Dry Rice husk-based powders, DRHP)

	Cu	Zn	Cd
RHF	0	108	1.5
RH-SCB	127.5	821	5.5
RHB	0	147.5	2.5

Table S6 The total mass of heavy metals in sludge filtrates and sludge cakes (mg)

		Raw sludge	Sludge conditioned with $FeCl_3$ (115.07 g/kg)	Sludge conditioned with $FeCl_3$ (115.07 g/kg) & RHF (70% DS)	Sludge conditioned with $FeCl_3$ (115.07 g/kg) & RHB (60% DS)	Sludge conditioned with $FeCl_3$ (115.07 g/kg) & RH-SCB (70% DS)
In sludge filtrate	Cu	0.00000	0.00000	0.00000	0.00000	0.00000
	Zn	0.10264	0.11380	0.09114	0.00420	0.05940
	Cd	0.00026	0.00028	0.00020	0.00011	0.00033
In sludge cake	Cu	0.12922	0.12913	0.12904	0.12943	0.21996
	Zn	0.54406	0.53129	0.61877	0.71610	1.15284
	Cd	0.00510	0.00498	0.00622	0.00698	0.00874
In rice husk-based powders	Cu	-	-	0.00000	0.00000	0.09051
	Zn	-	-	0.06427	0.07523	0.58283
	Cd	-	-	0.00106	0.00175	0.00390
Experimental values	Cu	0.12922	0.12913	0.12904	0.12943	0.21996
	Zn	0.64670	0.64509	0.70991	0.72030	1.21224
	Cd	0.00536	0.00526	0.00641	0.00708	0.00907
Theoretical values	Cu	0.12922	0.12922	0.12922	0.12922	0.21973
	Zn	0.64670	0.64670	0.71097	0.72193	1.22953
	Cd	0.00536	0.00536	0.00642	0.00711	0.00927

The theoretical values were equal to the total mass of heavy metals in the raw sludge and rice husk-based powders added. The values in Table S6 were average values, and the difference between theoretical and experimental values was caused by experimental errors.

Table S7 The RSP value of raw sludge and sludge cake conditioned with different Rice husk-based powders (ratio of secondary phase and primary phase, RSP)

	Cu	Zn	Cd
Raw sludge	43.05	144.00	7.25
$FeCl_3$ alone	48.07	173.88	8.01
RHF	25.74	27.74	5.35
RH-SCB	7.90	14.63	4.27
RHB	4.45	13.18	3.40

Table S8 Prices and parameters for economic analysis

Parameter	Unit	Value	Reference
$FeCl_3$ [a]	USD/kg	0.8	
CaO [a]	USD/kg	0.3	
Rise husk [a]	USD/kg	0.28	
RH-SCB	USD/kg	0.04	(Shahbeig and Nosrati, 2020)
Electricity price	USD/kWh	0.11	(Acien et al., 2012)
Sludge transport price [b]	USD/t	4.2	
Sludge disposal price [b]	USD/t	70.44	
Moisture content of rise husk	%	10.28	
Rise husk biochar yield	%	38.6	

[a] www.alibaba.com

[b] The price comes from some environmental protection enterprises in China

Reference

Acien, F.G., Fernandez, J.M., Magan, J.J., Molina, E., 2012. Production cost of a real microalgae production plant and strategies to reduce it. Biotechnol Adv 30, 1344-1353. https://doi.org/10.1016/j.biotechadv.2012.02.005

Gil-Lalaguna, N., Sanchez, J.L., Murillo, M.B., Atienza-Martinez, M., Gea, G., 2014. Energetic assessment of air-steam gasification of sewage sludge and of the integration of sewage sludge pyrolysis and air-steam gasification of char. Energy 76, 652-662. https://doi.org/10.1016/j.energy.2014.08.061

Li, R., Tang, C.Y., Li, X., Jiang, T., Shi, Y.P., Cao, Y.J., 2019. Reconstructing the historical pollution levels and ecological risks over the past sixty years in sediments of the Beijiang River, South China. Science of the Total Environment 649, 448-460. https://doi.org/10.1016/j.scitotenv.2018.08.283

Manganaro, J., Chen, B., Adeosun, J., Lakhapatri, S., Favetta, D., Lawal, A., Farrauto, R., Dorazio, L., Rosse, D.J., 2011. Conversion of Residual Biomass into Liquid Transportation Fuel: An Energy Analysis. Energy & Fuels 25, 2711-2720. https://doi.org/10.1021/ef200327e

Shahbeig, H., Nosrati, M., 2020. Pyrolysis of municipal sewage sludge for bioenergy production: Thermo-kinetic studies, evolved gas analysis, and techno-socio-economic assessment. Renewable and Sustainable Energy Reviews 119. https://doi.org/10.1016/j.rser.2019.109567
